# Correlation Study Between Clinical Special Tests for Myelopathy and Static MRI Parameters in Patients of Malaysian Population Treated for Cervical Dysfunction

**DOI:** 10.7759/cureus.18826

**Published:** 2021-10-16

**Authors:** Zamzuri Zakaria@Mohamad, Mohd. Ariff Sharifudin, Hishamudin Din, Azian Abd Aziz, Rajandra Kumar Karupiah

**Affiliations:** 1 Orthopaedics, Traumatology & Rehabilitation, International Islamic University Malaysia, Kuantan, MYS; 2 Orthopaedics and Traumatology, Universiti Sultan Zainal Abidin, Kuala Terengganu, MYS; 3 Radiology, International Islamic University Malaysia, Kuantan, MYS

**Keywords:** pathoanatomy, morphometric analysis, cord compression, cervical spondylosis, cervical spine

## Abstract

Introduction: Cervical spondylotic myelopathy (CSM) is the most common cause of spinal cord dysfunction. Magnetic resonance imaging (MRI) remains the imaging modality of choice, but its findings are not completely specific for clinically significant CSM. This cross-sectional study aims to determine the pathoanatomy of CSM in patients and analyze the correlations between clinical key symptoms, myelopathic signs, and MRI findings.

Methods: Patients with CSM aged 30 to 80 years old with no previous cervical spine disease or injuries were recruited. Clinical parameters include myelopathic hand signs and other clinical-specific tests. The MRI findings were analyzed for level of compression, underlying degenerative pathology, and parameters for cord compression.

Results: Thirty patients were recruited. The most common myelopathic signs observed were positive Hoffmann’s sign and the presence of reverse brachioradialis reflex. All patients had either degenerative or prolapse disc changes on MRI. There was a positive correlation between the clinical key features with MRI parameters for canal and cord diameter. The transverse cord diameter, cord compression ratio, and approximate cord area were the only independent variables related to almost all the positive clinical specific tests. All three have a moderate to strong correlation with the clinical findings.

Conclusion: The MRI parameters such as canal and cord size of the cervical spine are an objective reﬂection of compression on the spinal cord. Correlations observed indicate cord compression that plays a major role in the pathophysiology of CSM. These measurements are sensitive indicators of canal stenosis and play a signiﬁcant role in predicting the severity and outcome of CSM.

## Introduction

Cervical spondylotic myelopathy (CSM) is a non-specific degenerative process of the spine, which may result in varying degrees of stenosis of the cervical spinal canal [1-3]. It is the most common cause of cervical spinal cord dysfunction in middle-aged and older individuals [1-7]. However, some degree of clinical or radiological manifestation of CSM can manifest in individuals as young as 30 to 40 years old [8]. A normal cervical spine can uniquely adapt to allow a wide range of motions without compromising the spinal cord or the nerve roots [3]. The spinal cord may be compromised with the presence of degenerative changes leading to narrowing of the spinal canal including degenerative disc, osteophyte formations, and hypertrophy of lamina, articular facets, ligamentum flavum, and ossification of the posterior longitudinal ligament (OPLL) [1,4,9-11]. The incidence of degenerative changes differs between populations. For example, hypertrophied posterior ligamentum flavum was reported higher among the Japanese compared to other populations [10]. Limited studies on the pathoanatomy of CSM in the Malaysian population are available in the literature.

Cervical spondylotic myelopathy encompasses a range of symptoms and examination findings include motor and sensory abnormalities related to dysfunction of the cervical spinal cord. Patients usually present with gait disturbances, upper extremity weakness and impaired dexterity, lower extremity dysfunction or spasticity, and sexual dysfunction [2-4,7]. About 15 to 50% of patients also presented with bowel and bladder instability [3,12]. Several studies suggested gait disturbances following progressive weakness of upper extremities as the most common complaint among patients [13-15]. Occasionally, patients may also complain of neck stiffness in advanced cases of cervical spondylitis [2,7,15]. It can also manifest as one of the five cord syndromes depending on the extent of the condition and anatomical location of the cord damage [2,3]. Commonly affected levels are the C5 to C7 region of the cord [3-15].

The MRI remains the imaging modality of choice for CSM because of its superiority in evaluating neural structural pathology [3,4,12,16]. Associations between morphologic changes of the compressed spinal cord or the diameter of the spinal canal, and clinical symptoms of patients have been among the subject of interest among researchers [16]. Yu et al. reported a strong correlation between the degree of cord deformity on computed tomographic (CT) myelography and the severity of the symptoms [17]. However, the correlation between the degree of spinal cord compression on MRI findings and the extent of spinal cord dysfunction is not known [9,15]. This study was carried out to determine the correlation between the clinical special test for myelopathy and MRI findings in patients with cervical dysfunction.

## Materials and methods

This cross-sectional study was performed by reviewing patients diagnosed with CSM and their clinical and radiological records in a single tertiary center. The diagnosis of CSM was made based on the criteria by Cook et al. [18]. Inclusion criteria of patients recruited were aged between 30 and 80 years and had undergone an MRI evaluation less than one year after symptoms and signs were detected. Patients with congenital or developmental stenosis, patients who had an underlying metabolic disorder or previous cervical trauma, surgery, infection, or malignancy were excluded from the study. The study was commenced following the institutional research ethics committee approval (IIUM/305/14/11/3/IREC289).

The presence of specific clinical myelopathic signs namely Hoffmann’s sign, inverted supinator reflex, biceps reflex, scapulohumeral reflex, finger escape sign, grip and release test (10-second test), apparent ataxia, clonus, and Babinski reflex were recorded. Other clinical data documented were the modified Japanese Orthopaedic Association (mJOA) score (Table 1) [4,14], presence of clinical key symptoms including gait disturbances, upper limb clumsiness, bowel and urinary instability, sexual dysfunction, lower limb weakness, and upper limb sensory dysfunction. Motor power grades were based on the Medical Research Council (MRC) grading.

**Table 1:** Modification of the Japanese Orthopedic Association scoring system (mJOA score) by Benzel et al. [14]

Category	Score	Description
Motor dysfunction		
Upper extremity	0	Unable to move hand
	1	Unable to eat with a spoon but able to move hands
	2	Unable to button shirt but able to eat with a spoon
	3	Able to button shirt but with great difficulty
	4	Able to button shirt but with slight difficulty
	5	No dysfunction
Lower extremity	0	Complete loss of motor and sensory function
	1	Sensory preservation without the ability to move legs
	2	Able to move legs but unable to walk
	3	Able to walk on a flat floor with a walking aid
	4	Able to walk upstairs and/ or downstairs with the aid of a handrail
	5	Moderate-to-significant lack of stability but able to walk up and/ or downstairs without a handrail
	6	Mild lack of stability, but able to walk unaided with smooth reciprocation
	7	No dysfunction
Sensory dysfunction		
Upper extremity	0	Complete loss of hand sensation
	1	Severe sensory loss or pain
	2	Mild sensory loss
	3	No sensory loss
Sphincter dysfunction	0	Unable to micturate voluntarily
	1	Marked difficulty in micturition
	2	Mild-to-moderate difficulty in micturition
	3	Normal micturition

All the patients’ MRIs of the cervical spine, particularly the T2-weighted images, were evaluated and measured by a senior radiology consultant and a senior orthopaedic consultant surgeon. The measurements were performed mid-sagittal at each level of the cervical spine from C3 to C7 in millimeters. Each measurement was repeated three times by both consultants, and the mean of the measurements was calculated and taken as the final value for analyses. These include sagittal spinal canal diameter (SSCD), transverse spinal canal diameter (TSCD), anteroposterior cord diameter (APCD), and transverse cord diameter (TCD).

**Figure 1:**
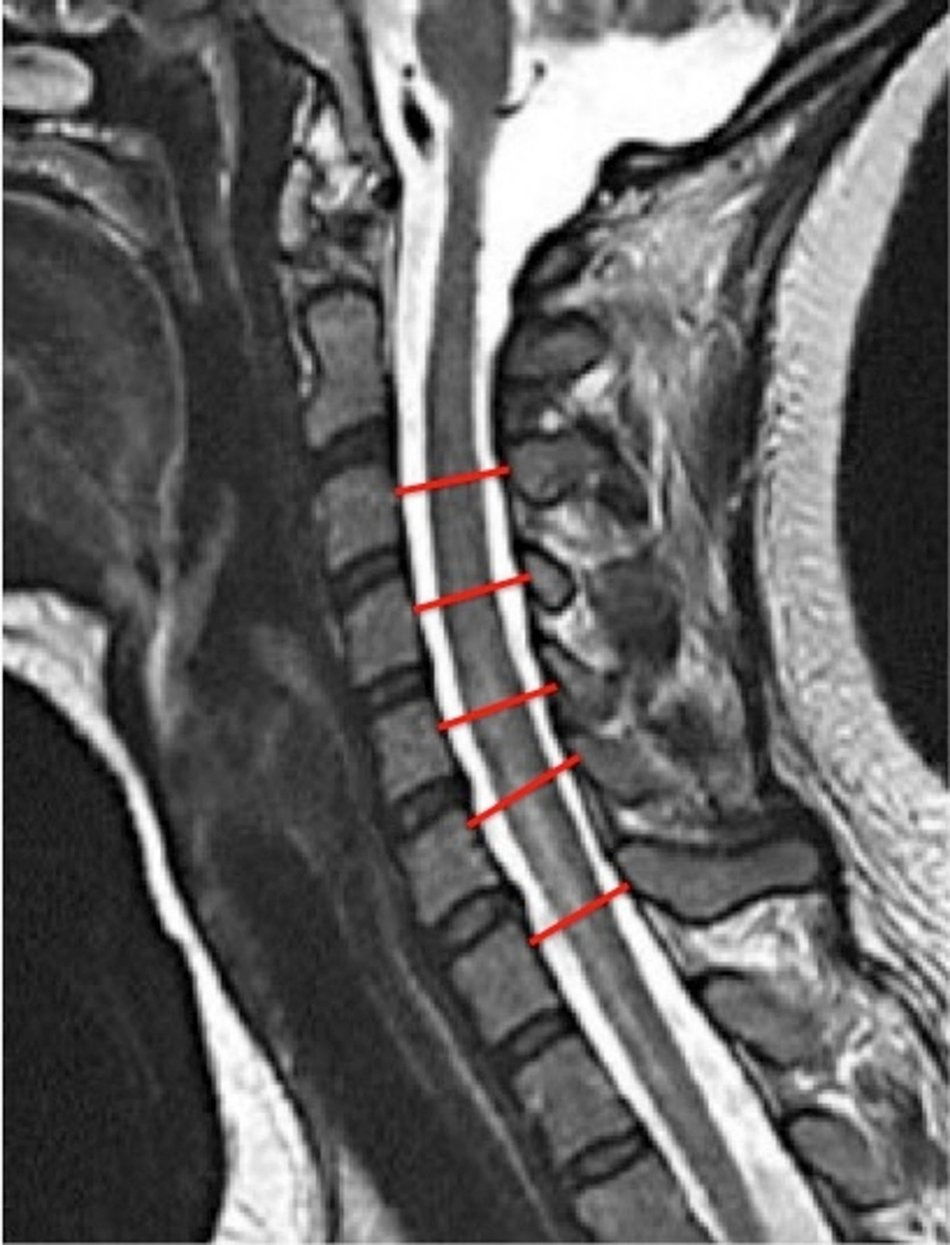
Measurement of sagittal spinal canal diameter (SSCD). SSCD of C3 to C7 (represented by the red lines) measured as the shortest distance from the midpoint between the vertebral body's superior and inferior endplates to the spinolaminar line of the corresponding vertebra body.

**Figure 2:**
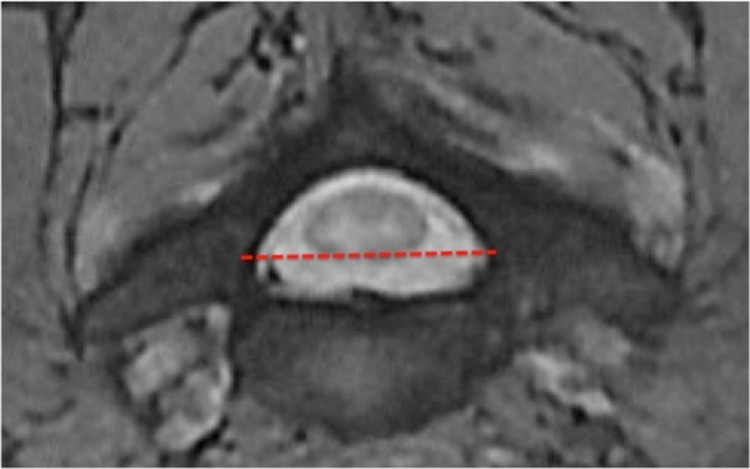
Measurement of transverse spinal canal diameter (TSCD) of the cervical vertebra as depicted by the dotted red line.

**Figure 3:**
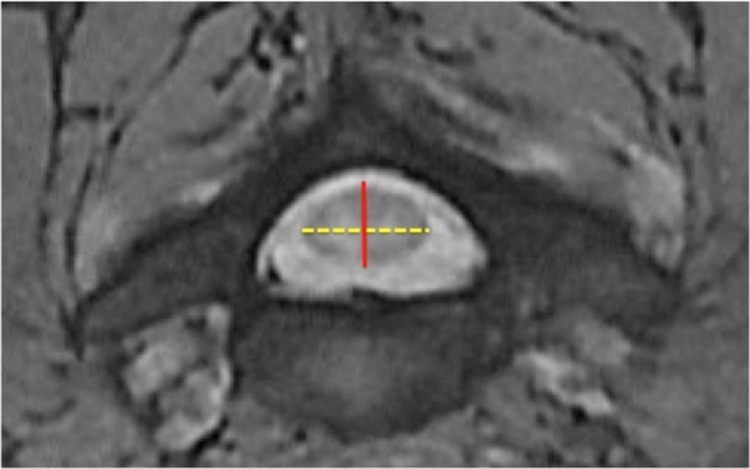
Measurement of anteroposterior cord diameter (APCD), and transverse cord diameter (TCD), as depicted by the red line and dotted yellow line, respectively. Measurements were taken at the corresponding disc level on the axial view.

The SSCD was measured as the shortest distance from the midpoint between the vertebral body's superior and inferior endplates to the spinolaminar line of the corresponding vertebral body (Figure 1). The TSCD was measured as the largest inner border inter-pedicle diameter of the corresponding cervical vertebra (Figure 2). The APCD and TCD measurements were taken at the corresponding disc level on the axial view (Figure 3). The true cord area was not calculated since the calculation involves variable elliptical shapes. However, a value termed the approximate cord area (ACA) was obtained as the simple measurement of the APCD and transverse diameters rounded to the nearest millimeter. Compression ratios (CR) were determined by APCD divided by TCD.

The presence of degenerative changes such as degenerated disc or protrusion of disc, osteophytes formation, facet hypertrophy, thickened or infoldings of ligamentum flavum, and OPLL was recorded. Measurement on each patient’s MRI was made from the digital images obtained from the hospital archives. Two investigators (a senior radiology consultant and a senior orthopedic consultant surgeon) performed the MRI evaluation and measurements for intra-observer and inter-observer reliability.

Statistical analyses of parametric data were performed using descriptive analysis (mean, median, and standard deviation) and independent t-score. The correlation was analyzed using Pearson correlation ratio and one-way analysis of variance (ANOVA) for numerical variables, and Spearman correlation for categorical data with SPSS software version 22.0 (IBM Corp., Armonk NY, USA). Statistical significance was taken at p<0.05.

## Results

A total of 30 patients were included. The mean age of the entire cohort was 51.8 years (range, 33 to 69 years). Seventy percent (n = 21) of the patients were male. A higher incidence of CSM occurred in Malays (80.0%), while the remaining were Chinese. The majority of patients had moderate (n = 17) and mild (n = 12) myelopathic symptoms, according to their mJOA scoring.

Ten patients (33.3%) had mild gait disturbance and were still able to walk unaided with smooth reciprocation, while 30% (n = 9) had moderate to significant gait disturbance but were still able to walk upstairs or downstairs without a handrail. Only 13.3% (n = 4) of patients were able to walk upstairs with the aid of a handrail. Ten percent of the patients (n = 3) were only able to walk on a flat floor with the aid of walking aid. An equal number of patients (n = 3) had no lower limb dysfunction and preservation of sensory function without the ability to move their leg. Forty percent of patients (n = 12) could not button their shirts but were still able to use a spoon for feeding. Eight (26%) had slight difficulty in buttoning their shirt and 20% of patients (n = 6) were still able to button their shirt but with great difficulty. Only 13.3% of them (n = 4) had a normal motor function. Most of the patients (93.3%) had mild upper limb sensory loss and the remaining 6.7% (n = 2) had severe sensory loss over the hand. More than half of the patients (53.3%) had mild to moderate difficulty in micturition, while 43% of them (n = 13) had no micturition problem. Only one patient had marked difficulty in micturition. Lower limb motor power was grade based on MRC motor power grading. Most of the patients (56.67%) had lower limb motor power of MRC 4. Patients with normal motor power make up 33.33% (n = 10) of this study population and the remaining 10% (n = 3) had muscle power of MRC 3. No patient with severe sexual dysfunction was found in this study. Sixty percent of patients had positive Hoffman’s sign. A similar percentage of patients (60%) had reverse supinator reflex, 26.7% had impaired 10-second test, and 20% showed a positive finger escape sign. 36.7% of patients were observed to have hyperreflexia of biceps jerk while 10% had appearance ataxia. 43.3% had sustained clonus while only 20% of patients were observed to have positive Babinski reflex . These findings are summarized in Table 2.

**Table 2:** Demographic characteristics with descriptive statistics of the severity of myelopathic symptoms and positive clinical special tests among patients (n = 30) SD: Standard Deviation

Variable	n (%)	Mean (SD)
Age (years)		51.80 (9.87)
30-40	5 (16.67)	
41-50	6 (20.00)	
51-60	13 (43.33)	
61-70	6 (20.00)	
Sex		
Male	21 (70.00)	
Female	9 (30.00)	
Ethnicity		
Malay	24 (80.00)	
Chinese	6 (20.00)	
Severity of myelopathic symptoms (based on mJOA score)		
Mild	12 (40.00)	
Moderate	16 (53.33)	
Severe	2 (6.67)	
Positive clinical special tests		
Hoffmann’s sign	18 (60.00)	
Grip and release test (Ten-second test)	8 (26.67)	
Finger escape sign	6 (20.00)	
Reverse brachioradialis reflex	18 (60.00)	
Biceps reflex	11 (36.67)	
Scapulohumeral reflex	9 (30.00)	
Apparent ataxia	3 (10.00)	
Sustained clonus	13 (43.33)	
Babinski reflex	6 (20.00)	

All patients identified to have either degenerative or prolapse disc changes in their MRI with 90% of them showing evidence of osteophyte formation. Facet hypertrophy changes at least at one level were seen in 36.7 % (n = 11) and 23.3% (n = 7) had thickening or infoldings of ligamentum flavum. None were identified with OPLL or lamina hypertrophy. The SSCD ranged from 11.20mm to 18.20mm. Mean SSCD was 15.53mm (Standard Deviation [SD] = 1.31). The SSCD was greatest at C7, and the smallest measured at the C4 vertebra. The TSCD ranged from 24.00mm to 28.10mm with a mean of 26.18mm (SD = 0.67). The TSCD increases exponentially from C3 to the C7 vertebra. The APCD ranged from 5.7mm to 9.3mm (mean of 7.34mm (SD = 0.32) with the largest measurement obtained at the C7 level and the smallest at the C5 level. The TCD ranged from 9.6mm to 12.6mm with a mean of 11.21mm (SD = 0.61). Similar to TSCD, TCD also increased exponentially from C3 to C7. Detailed measurements of the cervical canal and cord are summarized in Table 3.

**Table 3:** Detailed measurements of the cervical spinal canal and cord from C3 to C7 vertebra SD: Standard deviation

Parameters	Vertebrae level	Mean	SD	Minimum	Maximum
Transverse spinal canal diameter (TSCD)	C3	25.87	0.93	24.00	27.90
	C4	25.87	0.93	24.00	27.90
	C5	25.95	0.97	24.30	28.10
	C6	26.21	0.76	24.10	27.20
	C7	26.42	0.68	24.00	27.20
Sagittal spinal canal diameter (SSCD)	C3	15.21	1.12	12.80	16.80
	C4	15.17	1.23	12.00	17.10
	C5	15.50	1.33	11.30	17.10
	C6	15.68	1.37	11.20	17.40
	C7	16.19	1.50	11.30	18.20
Compression ratio (CR)	C3	0.69	0.04	0.59	0.76
	C4	0.66	0.04	0.56	0.75
	C5	0.58	0.07	0.47	0.74
	C6	0.65	0.06	0.51	0.74
	C7	0.70	0.07	0.56	0.85
Anteroposterior cord diameter (APCD)	C3	7.27	0.22	6.80	7.80
	C4	7.22	0.17	6.90	7.60
	C5	6.56	0.63	5.70	8.00
	C6	7.47	0.73	6.00	8.60
	C7	8.19	0.73	6.40	9.30
Transverse cord diameter (TCD)	C3	10.55	0.62	9.60	11.80
	C4	10.93	0.67	9.80	12.20
	C5	11.28	0.64	10.00	12.40
	C6	11.55	0.61	10.40	12.60
	C7	11.72	0.52	10.60	12.40
Approximate cord area (ACA)	C3	76.71	5.30	66.24	87.00
	C4	78.92	4.92	68.60	89.06
	C5	73.89	6.70	62.06	87.36
	C6	86.43	10.68	65.10	105.40
	C7	96.05	10.14	71.28	111.60

Correlations between MRI findings and clinical key symptoms in patients

One-way ANOVA showed there was a significant difference between the mean of patients with mild, moderate, and severe myelopathy scores in their SSCD (p<0.031), APCD (p<0.046), TCD (p<0.001), and ACA (p<0.001). The TCD, CR, and ACA were found significantly correlated across the entire clinical key symptoms. Upper limb sensory dysfunction did not correlate with any of the MRI parameters. All significant correlations showed a positive correlation except compression ratio, where it was shown to have a negative correlation. This means patients with smaller MRI parameters will have more severe myelopathy symptoms compared to patients with larger MRI parameters. Patients with a high compression ratio were more likely to have a severe degree of cervical myelopathy evidenced by the negative correlation found. The correlation of clinical key features and cervical MRI measurement parameters was analyzed using Pearson correlation analysis. There were significant correlations between patients’ clinical key features with the MRI parameters. These are listed in Table 4.

**Table 4:** Correlation between clinical key symptoms and MRI parameters

Clinical key symptoms	MRI parameters	Pearson correlation coefficient (r)	P-value
Gait disturbance	Sagittal spinal canal diameter (SSCD)	0.59	0.001
	Transverse spinal canal diameter (TSCD)	0.27	0.153
	Compression ratio (CR)	-0.34	0.065
	Anteroposterior cord diameter (APCD)	0.37	0.042
	Transverse cord diameter (TCD)	0.74	<0.001
	Approximate cord area (ACA)	0.77	<0.001
Upper limb clumsiness	Sagittal spinal canal diameter (SSCD)	0.52	0.003
	Transverse spinal canal diameter (TSCD)	0.07	0.720
	Compression ratio (CR)	-0.55	0.002
	Anteroposterior cord diameter (APCD)	0.08	0.688
	Transverse cord diameter (TCD)	0.75	<0.001
	Approximate cord area (ACA)	0.60	<0.001
Bowel and urinary instability	Sagittal spinal canal diameter (SSCD)	0.56	0.001
	Transverse spinal canal diameter (TSCD)	-0.08	0.658
	Compression ratio (CR)	-0.42	0.023
	Anteroposterior cord diameter (APCD)	0.04	0.820
	Transverse cord diameter (TCD)	0.56	0.001
	Approximate cord area (ACA)	0.40	0.014
Sexual dysfunction	Sagittal spinal canal diameter (SSCD)	0.31	0.050
	Transverse spinal canal diameter (TSCD)	-0.03	0.890
	Compression ratio (CR)	-0.49	0.005
	Anteroposterior cord diameter (APCD)	-0.15	0.420
	Transverse cord diameter (TCD)	0.49	0.007
	Approximate cord area (ACA)	0.27	0.156
Lower limb weakness	Sagittal spinal canal diameter (SSCD)	0.46	0.024
	Transverse spinal canal diameter (TSCD)	0.12	0.530
	Compression ratio (CR)	-0.45	0.013
	Anteroposterior cord diameter (APCD)	0.13	0.490
	Transverse cord diameter (TCD)	0.67	<0.001
	Approximate cord area (ACA)	0.57	<0.001
Upper limb sensory dysfunction	Sagittal spinal canal diameter (SSCD)	0.14	0.457
	Transverse spinal canal diameter (TSCD)	-0.31	0.871
	Compression ratio (CR)	-0.26	0.174
	Anteroposterior cord diameter (APCD)	-0.33	0.077
	Transverse cord diameter (TCD)	0.58	0.761
	Approximate cord area (ACA)	-0.16	0.396

Correlations between MRI findings and specific clinical myelopathic signs

Based on analyses with an independent t-test, presences of specific clinical signs were related to some of the MRI measurement parameters. The parameters were also found significantly correlated with the specific clinical signs after being analyzed with the Spearman correlation test. Details of the correlations and their respective p-value are summarized in Table 5. Using the Spearman correlation test, SSCD, TCD, and ACA were found to have a significant correlation across the entire clinical myelopathic signs. Strong to moderate positive correlations were observed in all significant parameters. In other words, patients with smaller MRI parameters will have more positive specific clinical myelopathic signs compared to patients with larger MRI parameters.

**Table 5:** Correlation between specific clinical myelopathic signs and MRI parameters

Specific clinical myelopathic signs	MRI parameters	Spearman correlation coefficient (r)	P-value
Hoffmann's sign	Sagittal spinal canal diameter (SSCD)	0.45	0.014
	Transverse spinal canal diameter (TSCD)	-0.04	0.847
	Compression ratio (CR)	-0.59	0.001
	Anteroposterior cord diameter (APCD)	-0.02	0.917
	Transverse cord diameter (TCD)	0.71	<0.001
	Approximate cord area (ACA)	0.52	0.003
Inverted supinator reflex	Sagittal spinal canal diameter (SSCD)	0.52	0.003
	Transverse spinal canal diameter (TSCD)	-0.03	0.897
	Compression ratio (CR)	-0.56	0.001
	Anteroposterior cord diameter (APCD)	0.09	0.645
	Transverse cord diameter (TCD)	0.07	<0.001
	Approximate cord area (ACA)	0.6	<0.001
Bicep's reflex	Sagittal spinal canal diameter (SSCD)	0.57	0.001
	Transverse spinal canal diameter (TSCD)	0.26	0.164
	Compression ratio (CR)	-0.37	0.043
	Anteroposterior cord diameter (APCD)	0.3	0.108
	Transverse cord diameter (TCD)	0.7	<0.001
	Approximate cord area (ACA)	0.7	<0.001
Scapulohumeral reflex	Sagittal spinal canal diameter (SSCD)	0.6	<0.001
	Transverse spinal canal diameter (TSCD)	0.35	0.060
	Compression ratio (CR)	-0.27	0.157
	Anteroposterior cord diameter (APCD)	0.4	0.028
	Transverse cord diameter (TCD)	0.66	<0.001
	Approximate cord area (ACA)	0.74	<0.001
Finger escape sign	Sagittal spinal canal diameter (SSCD)	0.70	<0.001
	Transverse spinal canal diameter (TSCD)	0.29	0.119
	Compression ratio (CR)	-0.36	0.053
	Anteroposterior cord diameter (APCD)	0.31	0.096
	Transverse cord diameter (TCD)	0.71	<0.001
	Approximate cord area (ACA)	0.52	0.003
Grip and release test (10-second test)	Sagittal spinal canal diameter (SSCD)	0.67	<0.001
	Transverse spinal canal diameter (TSCD)	0.35	0.059
	Compression ratio (CR)	-0.26	0.163
	Anteroposterior cord diameter (APCD)	0.48	0.007
	Transverse cord diameter (TCD)	0.72	<0.001
	Approximate cord area (ACA)	0.83	<0.001
Apparent ataxia	Sagittal spinal canal diameter (SSCD)	0.42	0.021
	Transverse spinal canal diameter (TSCD)	0.5	0.005
	Compression ratio (CR)	-0.58	0.762
	Anteroposterior cord diameter (APCD)	0.6	0.001
	Transverse cord diameter (TCD)	0.42	0.021
	Approximate cord area (ACA)	0.67	<0.001
Clonus	Sagittal spinal canal diameter (SSCD)	0.57	0.001
	Transverse spinal canal diameter (TSCD)	0.12	0.526
	Compression ratio (CR)	-0.05	0.762
	Anteroposterior cord diameter (APCD)	0.18	0.348
	Transverse cord diameter (TCD)	0.76	<0.001
	Approximate cord area (ACA)	0.67	<0.001
Babinski reflex	Sagittal spinal canal diameter (SSCD)	0.63	<0.001
	Transverse spinal canal diameter (TSCD)	0.34	0.068
	Compression ratio (CR)	0.00	1.000
	Anteroposterior cord diameter (APCD)	0.50	0.005
	Transverse cord diameter (TCD)	0.46	0.011
	Approximate cord area (ACA)	0.65	<0.001

## Discussion

**Figure 4:**
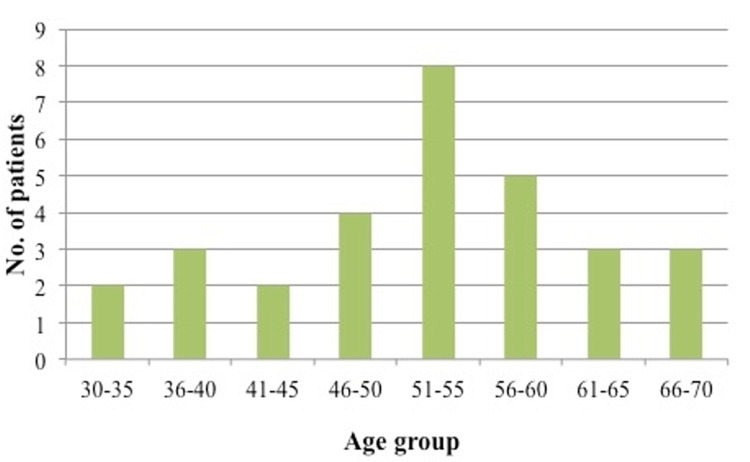
Bar chart showing the distribution of patients according to the age group

Cervical myelopathy is a relatively common condition affecting a spectrum of patients across different age groups. However, the epidemiology of CSM, including its incidence and prevalence, is underreported in Western literature [1,9]. On the other hand, it is quite a common disease within the Asian populations, particularly among the Chinese and Japanese [1,6,9,10]. Northover et al. concluded that CSM predominantly affects men in their seventh decade of life [5]. The study recruited a total of 41 patients with a mean age of 68.7 years from the United Kingdom. Kokubun et al. found that most of their patients were in their sixth or seventh decade of life [6]. Patients in the current study ranged from 33 to 69 years old with the majority of them from the 51- to 55-year-old age group (Figure 4). Most of them had mild to moderate myelopathic symptoms. We postulated that the earlier age of presentation of our patients was due to the rarity of elderly patients with severe myelopathy in our population seeking medical intervention and opted for traditional treatment. Occasionally, their conditions were misdiagnosed as other medical problems and were not referred to the orthopedic specialist for assessment. This is also one of the reasons why the incidence of severe myelopathy is very low in this study. A higher incidence of CSM occurs in Malays (80%) compared to Chinese patients and depicts our diverse general population with the Malays being the predominant ethnic group.

A degenerative cascade in the spinal cord that is commonly seen in CSM leads to direct compressive and ischemic dysfunction of the spinal cord [1,3,4,7,11,15,19,20]. Similar to other populations of the world, the main etiology of cervical spondylosis in our cohort of patients was the degenerative changes mainly due to disc degeneration and osteophyte formation that are both attributed to cervical spondylosis. None of the patients’ myelopathy was attributed to OPLL. Ossification of the posterior longitudinal ligament is reported mostly in literature from Japan, where prevalence rates are higher compared with other ethnic groups [1,21]. In Taiwan and Korea, the rate in the population is near 3% [22]. As there are racial differences in the incidence of OPLL, one of the etiologies is thought to be environment and lifestyle, including diet.

The pathophysiology of CSM involves static factors that cause stenosis, and dynamic factors resulting in repetitive injury to the spinal cord [9,11,16,19,23]. The size of the spinal cord has been postulated as one of the important factors in the development and progression of CSM [23]. The clinical presentations of this disorder are generally related to the degree of compression of the various spinal cord tracts [11]. Dynamic MRI studies in patients with CSM show a significant increase of spinal stenosis that has been observed in extension more so than in flexion [16,19,23]. 'Absolute stenosis' has been defined as a sagittal canal diameter <10mm, and 'relative stenosis' as a canal diameter <13mm, and a normal sagittal diameter in the mid-cervical spine of 17 to 18mm [23]. However, these absolute measurements of sagittal plane diameter are subject to genetic variation between individuals of different sizes. The sagittal diameter of the spinal cord is nearly constant in adults averaging approximately 8mm from C3 to C7 [24].

In the current study, we found the average sagittal canal diameter from C3 to C7 to be 15.55 ± 1.27mm and the average sagittal cord diameter to be 7.34 ± 0.32mm. These findings are larger compared to other previous literature [16,25]. Although our average sagittal canal diameter did not fall into the group of relative stenosis, they already show evidence of myelopathy sign as most of the stenotic level in our patients occurs at one or two levels only, thus the average sagittal canal diameter is not a true reflection of cervical spinal stenosis in general. We believe this finding brings in additional information for further study and exploration. Furthermore, the measured parameter was based on static MRI, which is not a true representative of stenosis for a reason that had been explained earlier. Gu et al. suggest that cervical myelopathic patients, in general, have narrower spinal canals shown on cervical spinal radiographs, and patients with higher baseline Japanese Orthopaedic Association (JOA) scores were observed to have a larger transverse cross-sectional area (TCSA) of the spinal cord compared to patients with lower JOA [26]. Hence, the symptoms of this disorder are generally related to the degree of compression of the various spinal cord tracts.

The transverse area of the cord measured using MRI has been reported to have a strong correlation with the severity of myelopathy, the pathological changes in the cord, and post-surgical recovery of patients with cervical compressive myelopathy [16,27]. Cadaveric studies have shown that the TCSA of the spinal cord and the CR of the spinal cord correlate with the severity of pathologic changes [20,27]. This finding was found to be true in the current study, as TCD and CR have mild to moderate correlation with clinical key features. The phrase ‘uppers in the lowers and lowers in the uppers’ simply summarizes the physical signs of cervical myelopathy [7]. In other words, patients with cervical myelopathy will exhibit upper motor neuron signs distal to compression in the upper and lower limbs lower motor neuron manifestations in the upper limbs at the level of compression are observed [15]. Myelopathy hand signs, a group of characteristic dysfunctions of the hand, were reportedly attributed to reflex disinhibition and pyramidal tract involvement following the pathological changes in the cervical spine [15]. Although no correlation has been reported between the hand myelopathy signs and the level of cervical cord involvement, these signs are frequently observed at the level of compression above C5/C6, but uncommonly seen at C6/C7 level and below [15].

The myelopathic hand signs and other specific clinical signs were found significantly correlated with cervical cord compression, and significantly observed in our patients with smaller cord diameters. Wong et al., however, reported no correlation between the presence of myelopathic hand signs and radiological assessment or cord diameter [9]. There is a significant correlation between the clinical key features with most of the MRI parameters. The TCD, CR, and ACA were found to be the only independent variables related to almost all the clinical key features in the presence of myelopathy, except in hand sensory dysfunction, sexual dysfunction, and lower limb dysfunction. These exceptions concur with some of the findings by Coronado et al. [13]. All three parameters have moderate to strong correlations with clinical key features. Compression ratios have a significant negative correlation with most clinical key features, except lower limb dysfunction and sensory dysfunction of the upper limb, compared to a positive correlation with TCD and ACA. All these correlations indicate that cord compression plays a major role in the pathophysiology of CSM. These measurements can be considered as sensitive indicators of canal stenosis. Using the current investigation as a pilot study, further research using a larger patient population may help to further clarify the findings of this study and elucidate the details regarding the relationship and correlation between cervical spinal canal and spinal cord diameter on the risk of myelopathy. New scoring systems can be devised allowing prognostication of the severity of symptoms with the degree of cord compression and to assist surgeons in managing this condition.

This study was not without limitations. A sample size of 30 patients with an unequal number of male and female patients might not reflect the true representation of the entire population. The study only involved one tertiary center. Furthermore, dynamic MRI parameters would be better than static MRI. Nevertheless, this study serves as a foundation upon which to build future investigations, including additional data from future studies that can help improve the understanding of MRI predictors of disease severity and outcomes in patients with CSM.

## Conclusions

Cord compression plays a major role in the pathophysiology of CSM. The MRI parameters such as canal and cord size of the cervical spine can objectively reflect the compression on the spinal cord. These measurements can be utilized as sensitive indicators of canal stenosis as well as play a significant role in predicting the severity and outcome of CSM.
